# 

**Published:** 1938

**Authors:** 


### The Bristol Medico-Chirurgical Journal

A JOURNAL OF THE MEDICAL SCIENCES FOR THE WEST OF ENGLAND AND SOUTH WALES.

PUBLISHED UNDER THE AUSPICES OF

*THE BRISTOL MEDICO-CHIRURGICAL SOCIETY.*

EDITOR:

J. A. NIXON, C.M.G., M.D., F.R.C.P.

WITH WHOM ARE ASSOCIATED

E. W. HEY GROVES, D.Sc., M.S., F.R.C.S.
A. E. ILES, O.B.E., M.B., B.S., F.R.C.S.
A. RENDLE SHORT, M.D., B.S., B.Sc., F.R.C.S.

E. WATSON-WILLIAMS, M.C., Ch.M., F.R.C.S., Assistant Editor.

EDITORIAL SECRETARY:

A. L. FLEMMING, M.B., Ch.B., D.A.

---

"Scire est nescire, nisi id me

Scire alius sciret."

---

ARMY MEDICAL LIBRARY

335144

JUL 25 '40

WASHINGTON, D.C., U.S.A.

*VOL. LV.*

BRISTOL: J. W. ARROWSMITH LTD.
London: J. W. Arrowsmith (London) Ltd.

WI
BR323

### Contents of Vol. LV

*Page*

Medical Defence. By CLIFFORD A. MOORE, M.S., CH.M., F.R.C.S. .. 1

Some Clinical Applications of the Male and Female Sex Hormones. By G. L. FOSS, M.B., B.CH. .. .. .. .. .. .. 23

Thoracic Surgery. By A. L. D'ABREU, CH.M., F.R.C.S. .. .. 43

A Case of Lobectomy for Bronchiectasis. By A. WILFRID ADAMS, M.S., F.R.C.S. .. .. .. .. .. .. .. .. 63

Psychological Factors in Disease. By T. M. LING, B.M., B.CH., M.R.C.P. 101

Cerebral Malformations and their Clinical Consequences. By R. J. A. BERRY, M.D., F.R.C.S., F.R.S.E. .. .. .. .. .. 111

The Use of Protamine Insulin. By J. A. NIXON, C.M.G., M.D., F.R.C.P. 121

Some Problems in the Diagnosis and Treatment of Intra-Cranial Tumours. By F. WILFRED WILLWAY, M.D., M.S., B.SC., F.R.C.S. 151

Some Tuberculous Conditions. By HUBERT CHITTY, M.S., F.R.C.S. 161

The Vitamin B Complex. By H. M. SINCLAIR, M.A., B.SC., B.M., B.CH. 173

The Medicinal Waters at Burnham-on-Sea. By J. A. NIXON, C.M.G., M.D., F.R.C.P. .. .. .. .. .. .. .. .. 191

The Twenty-seventh Long Fox Memorial Lecture: Air as a Conductor of Electricity. By PROFESSOR A. M. TYNDALL, D.SC., F.R.S. .. 211

Syphilis in the Tropics. By T. F. HEWER, M.D., M.R.C.P. .. .. 217

A Guide Dog for the Blind. By F. WILFRED WILLWAY, F.R.C.S. 235

Drinker's "Iron Lung" and Other Artificial Respirators. By J. A. NIXON, C.M.G., M.D., F.R.C.P. .. .. .. .. .. 239

REVIEWS OF BOOKS .. .. .. .. .. .. 67, 132, 199, 245

EDITORIAL NOTES .. .. .. .. .. .. 78, 140, 203, 260

OBITUARIES .. .. .. .. .. .. .. 83, 145, 205, 262

MEETINGS OF SOCIETIES .. .. .. .. .. 85, 146, 267

THE MEDICAL LIBRARY OF THE UNIVERSITY OF BRISTOL 86, 146, 207, 270

PUBLICATIONS RECEIVED .. .. .. .. .. 90, 148, 209, 272

LOCAL MEDICAL NOTES .. .. .. .. .. 91, 149, 210, 273

### The Medical Library of the University of Bristol

WITH WHICH IS INCORPORATED

**The Library of the Bristol Medico-Chirurgical Society.**

---

*The following donations have been received since the publication of the list in January,* 1938.

April, 1938.

| | | |
|---|---|---|
| The Association of American Physicians (1) | .. | 1 volume. |
| Brompton Hospital (2) | .. .. .. .. | 1 volume. |
| Universidad de Buenos Aires (3) | .. .. .. | 2 volumes. |
| General Medical Council (4) | .. .. .. | 3 volumes. |
| University of Glasgow (5) | .. .. .. | 1 volume. |
| Professor E. W. Hey Groves (6) | .. .. .. | 3 volumes. |
| Michell Clarke Fund (7) | .. .. .. .. | 39 volumes. |
| Munro Smith Fund (8) | .. .. .. .. | 7 volumes. |
| Professor C. Bruce Perry (9) | .. .. .. | 2 volumes. |
| Professor A. Rendle Short (10) | .. .. .. | 1 volume. |
| Cyril H. Walker, Esq. (11) | .. .. .. | 1 volume. |

Unbound periodicals have also been received from Professor E. W. Hey Groves, Dr. T. Milling, Professor C. Bruce Perry and Dr. J. Odery Symes.

### THE ONE HUNDRED AND SIXTY-SECOND LIST OF BOOKS.

The figures in round brackets refer to the figures after the names of the donors. The books to which no such figures have been attached have either been bought from the Library Fund or received through the *Journal.*

| | | | |
|---|---|---|---|
| **Arce, José** .. .. .. | *Discursos del Decano de la Facultad de Ciencios Medicas, Universidad de Buenos Aires* .. .. .. .. .. .. .. .. .. | (3) | 1937 |
| **Barrington Ward, Sir L.** | *The Abdominal Surgery of Children* | 2nd Ed. | 1937 |
| **Beaumont, G. E.** .. .. | *Medicine* .. .. .. .. .. | 3rd Ed. (7) | 1937 |

Bickerton, T. H. .. .. *A Medical History of Liverpool* .. (11) 1936
Bigger, J. W. .. .. *Handbook of Hygiene* .. .. .. .. .. .. 1937
Bray, G. W. .. .. .. *Recent Advances in Allergy* 3rd Ed. (7) 1937
Brown, W. Langdon, and Hilton, R. *Physiological Principles in Treatment* .. .. .. .. .. 7th Ed. (7) 1936
Buchanan .. .. .. *Manual of Anatomy* .. .. 6th Ed. (7) 1937
Buckley, C. W. (Ed.) .. *Reports on Chronic Rheumatic Diseases,* No. 3 .. .. .. .. .. .. .. .. .. 1937
Bunting, R. W. .. .. *Oral Hygiene and the Treatment of Parodontal Diseases* .. .. .. (7) 1936
Charlesworth, F. .. .. *Chiropody—Theory and Practice* .. (8) 1935
Clark, A. J. .. .. .. *Applied Pharmacology* .. 6th Ed. (7) 1937
*Criteria for the Classification and Diagnosis of Heart Disease* 3rd Ed. (7) 1932
Cruickshank, E. W. H. *Food and Physical Fitness* .. .. .. .. .. 1937
Cunningham .. .. .. *Textbook of Anatomy* .. .. 7th Ed. (7) 1937
Cushny, A. R. .. .. *Textbook of Pharmacology and Therapeutics* 11th Ed. (7) 1936
Derham, S. .. .. .. *Hydrologia Philosophica* .. .. .. .. .. 1685
Doxtater, L. W. .. .. *Full and Partial Dental Prosthesis* .. (7) 1936
Edwards, H. C. .. .. *Surgical Emergencies in Children* .. .. 1936
Fairbrother, R. W. .. *A Textbook of Medical Bacteriology* .. 1937
Fearon, R. .. .. .. *Nutritional Factors in Disease* .. .. (7) 1936
Garcia, C. L. .. .. *Las Deformidades de la Sexualidad Humana* (3) 1925
Greenfield, A. L. .. .. *X-ray Technic and Interpretation of Dental Roentgenograms* .. .. .. .. (7) 1936
Gask, G. E., and Ross, J. Paterson. *Surgery of the Sympathetic Nervous System* .. .. 2nd Ed. (7) 1937
Hale-White, W. .. .. *Materia Medica, Pharmacy, Pharmacology and Therapeutics* .. .. 23rd Ed. (7) 1937
Halliburton, W. D., and McDowall, R. J. S. *Handbook of Physiology* 35th Ed. (7) 1937
Harris, L. .. .. .. *Vitamins in Theory and Practice* 2nd Ed. (7) 1937
Henning, N. .. .. .. *Textbook of Gastroscopy.* (Trans. H. W. Rodgers) .. .. .. .. .. .. .. .. .. 1937
Hewer, C. L. .. .. *Recent Advances in Anæsthesia and Analgesia* 2nd Ed. (7) 1937
Hill, A. Bradford .. *Principles of Medical Statistics* .. (7) 1937
Hillary, W. .. .. .. *An Inquiry into the Contents and Medicinal Virtues of Lincomb Spaw Water, near Bath* .. .. .. .. .. .. .. .. .. .. 1742
Hindley-Smith, J. D. .. *Chronic Streptococcal Infection as a Disease* 1937
Hutchison, R., and Mottram, V. H. *Food and the Principles of Dietetics* 8th Ed. (7) 1936
Ingleson, H. .. .. .. *The Action of Water on Lead.* (Water Pollution Research Technical Papers, No. 4) .. .. .. .. .. .. .. .. 1934

**Ling, T. M.** .. .. .. *Recent Advances in Industrial Hygiene and Medicine* .. .. .. .. .. .. .. (8) 1937

**Lōhr, W.** .. .. .. *Wundheilung* .. .. .. .. .. .. .. (6) 1937

**MacCullum, W. G.** .. *A Textbook on Pathology* .. 6th Ed. (7) 1937

**McKenny-Hughes, A. W.** *The Bed-bug.* (British Museum (Natural History) Economic Series, No. 5) .. .. 1937

**Manson, Sir P.** .. .. *Tropical Diseases* .. 10th Ed. (7) 1935

**Martindale, W. H.** .. *The Extra-pharmacopoeia.* Vol. 1 21st Ed. (7) 1936

**Meakins, J. C.** .. .. *The Practice of Medicine* .. .. .. (7) 1937

**Means, J. H.** .. .. *The Thyroid and its Diseases* .. .. (7) 1937

**Moncrieff, A.** .. .. *The Management of the New-born Baby* (7) 1936

**Muir, R.** .. .. .. *Textbook of Pathology* .. .. 4th Ed. (7) 1936

**Muir and Ritchie** .. *Manual of Bacteriology* .. 10th Ed. (7) 1937

**Oldfield, M. C.** .. .. *Speech Training for Cases of Cleft Palate* .. 1938

**Petersen, W. F.** .. .. *The Patient and the Weather.* Vol. IV. Part 3. *Organic Disease—Surgical Problems* .. .. .. .. .. .. .. .. .. 1938

**Poulton, E. P.** .. .. *Diets and Recipes—and the Treatment of Diabetes and Obesity* .. .. .. (9) 1937

**Prinz, H.** .. .. .. *Dental Formulary* .. .. .. .. (7) 1936

**Prinz, H.** .. .. .. *Diseases of the Soft Structures of the Teeth and their Treatment* .. 2nd Ed. (7) 1937

**Puech, Rezende** .. .. *O problema da luxacão congenita do quadril no Brasil* .. .. .. .. .. .. .. (6) 1937

**Purves-Stewart, Sir J.** *The Diagnosis of Nervous Diseases* 8th Ed. (7) 1937

*Queen Charlotte's Textbook of Obstetrics* .. .. .. .. .. 4th Ed. (7) 1937

*Radium, Production, General Properties, its Applications in Therapeutics* (10) 1937

**Romanis, W. H. C., and Mitchiner, P. H.** *The Science and Practice of Surgery.* 2 vols. .. .. 6th Ed. (8) 1937

**Rowlands, R. P., and Turner, P.** *The Operations of Surgery.* 2 vols. 8th Ed. (8) 1936

**Sauerbruch, F.** .. .. *Advances in Modern Surgery.* (University of Glasgow, MacEwen Memorial Lecture, 1937.) .. .. .. .. .. .. .. .. (5) 1937

**Sauerbruch, F., and O'Shaughnessy, L.** *Thoracic Surgery* .. .. (8) 1937

**Sheldon, W.** .. .. .. *Diseases of Infancy and Childhood* .. (7) 1936

**Sherman, H. C.** .. .. *Chemistry of Food Nutrition* 5th Ed. (7) 1937

**Simpson, W.** .. .. *Hydrological Essayes* .. .. .. .. .. .. 1670

**Skinner, E. W.** .. .. *The Science of Dental Materials* .. (7) 1937

**Smith, H. W.** .. .. *The Physiology of the Kidney* .. .. .. 1937

**Taylor, F.** .. .. .. *Practice of Medicine* .. .. 15th Ed. (7) 1936

**Thoma, K. H.** .. .. *Oral Diagnosis and Treatment Planning* (7) 1937

**Tweedy, E. H.** .. .. *Practical Obstetrics* .. .. .. 7th Ed. 1937

**Villafañe, I. Z.** .. .. *La funcion del periostio* .. .. .. (6) 1937

| | | | |
|---|---|---|---|
| **Warwick and Tunstall** | *First Aid to the Injured and Sick* | 16th Ed. | 1937 |
| **Whitby, L. E. H., and Britton, C. J. C.** | *Disorders of the Blood* | 2nd Ed. (7) | 1937 |
| **Williams** .. .. .. | *Obstetrics* .. .. .. .. .. .. | 7th Ed. (7) | 1936 |
| **Young, J.** .. .. .. | *Textbook of Gynæcology* .. | 4th Ed. (7) | 1936 |

TRANSACTIONS, REPORTS, JOURNALS, ETC.

| | | |
|---|---|---|
| L'Année Médicale Pratique. Vol. XVI. .. .. .. .. .. .. | (9) | 1937 |
| Brompton Hospital Reports. Vol. VI. .. .. .. .. .. .. .. .. | (2) | 1937 |
| The Dentists' Register, 1938 .. .. .. .. .. .. .. .. .. .. .. | (4) | 1938 |
| Medical and Dental Students Register, 1937 .. .. .. .. .. | (4) | 1938 |
| The Medical Register, 1938 .. .. .. .. .. .. .. .. .. .. .. | (4) | 1938 |
| Report on the 8th International Congress of Military Medicine and Pharmacy .. .. .. .. .. .. .. .. .. .. .. .. .. .. .. .. | | 1935 |
| St. Thomas's Hospital Reports. 1st Series, Vol. LIX, and 2nd Series, Vol. II. .. .. .. .. .. .. .. .. .. .. .. .. .. .. .. .. .. | | 1937 |
| Transactions of the Association of American Physicians. Vol. LII. | (1) | 1937 |

### NOTICE.

The following books have been missing from the Library for a long time, in spite of repeated requests for their return. Will members please be good enough to have a careful search made for these, in order that they may be returned without further delay ?

| | |
|---|---|
| **Bailey** .. .. .. .. | *Physical Signs in Clinical Surgery.* |
| **Berkeley** .. .. .. | *Handbook of Midwifery.* Ed. 9. 1935. |
| **Bourne** .. .. .. | *Synopsis of Obstetrics.* Ed. 6. |
| **Boyd** .. .. .. .. | *Research on Low Potencies of Homœopathy.* |
| **Byrne** .. .. .. .. | *Studies in the Physiology of the Eye.* |
| **Gadd** .. .. .. .. | *Synopsis of the Pharmacopœia.* |
| **Holmes** .. .. .. | *Cancer and Scientific Research.* |
| **Howard and Patry** .. | *Mental Health.* |
| **Hutton** .. .. .. | *The Last of the Taboos.* |
| **Knaus** .. .. .. | *Periodic Fertility and Sterility in Women.* |
| **Macalister** .. .. .. | *Narrative of an Investigation concerning an Ancient Medicinal Remedy.* |
| **Marshall** .. .. .. | *New Theory of Cancer.* |
| **Morris** .. .. .. | *Maternity and Post-operative Exercises.* |
| **Norman** .. .. .. | *Essentials of Modern Medical Treatment.* |
| **Sutherland** .. .. .. | *Tuberculin Handbook.* |
| **Walker** .. .. .. | *Status of Enzymes and Hormones in Therapy.* |
| **Whitnall** .. .. .. | *The Study of Anatomy.* Ed. 3. |

### Publications Received

From Messrs. J. W. Arrowsmith Ltd.:

*The Baths of Bath.* By P. Rowland James, M.A. Price 5s.

From Baillière, Tindall & Cox:

*Occupational Therapy. An Addendum to the Handbook for Mental Nurses.* Price 6d.

*Modern Treatment in General Practice.* Vol. IV. (1938). Edited by C. P. G. Wakeley, D.Sc., F.R.C.S., F.R.S.E., F.A.C.S., F.R.A.C.S. Price 10s. 6d.

From Edwards Brothers Inc., U.S.A.:

*The Patient and the Weather.* By William F. Petersen, M.D. Vol. IV. Part 3. *Organic Disease, Surgical Problems.* Price $10.

From Eyre and Spottiswoode Ltd.:

*Myocarditis.* The St. Cyres Memorial Lectures. Price 10s. 6d.

*Practical Procedures.* By Sir Humphrey Rolleston, Bart., G.C.V.O., K.C.B., M.D., F.R.C.P., and Alan A. Moncrieff, M.D., F.R.C.P. Price 10s. 6d.

From W. Heffer & Sons Ltd.:

*Surface and Radiological Anatomy.* By Arthur B. Appleton, M.A., M.D., William J. Hamilton, M.D., B.Ch., D.Sc., F.R.S.E., and Ivan C. C. Tchaperoff, M.A., M.D., B.Ch., D.M.R.E. Price 15s.

From William Heinemann Ltd.:

*Neuro-ophthalmology.* By R. Lindsay Rea, B.Sc., M.D., M.Ch., F.R.C.S. Price 42s.

From Messrs H. K. Lewis & Co. Ltd.:

*Studies on the Physiology of the Eye.* By J. Grandson Byrne, M.A., M.D., LL.D. Ed. 2. Price 40s.

*Studies on the Physiology of the Middle Ear.* By J. Grandson Byrne, M.A., M.D., LL.D. Price 18s.

*Speech Training for Cases of Cleft Palate.* By M. C. Oldfield, M.Ch. (Oxon), F.R.C.S. Price 4s. 6d.

*Notes on Bacteriology and Clinical Pathology for Nurses.* By Herbert Rogers, M.B., Ch.B. Price 1s.

*A Textbook of X-ray Diagnosis.* Edited by S. Cochrane Shanks, M.D., Peter Kerley, M.D., M.R.C.P., D.M.R.E., and E. W. Twining, M.R.C.S., L.R.C.P., D.M.R.E. By British authors. Vol. I. Price 50s. Vol. II. Price 42s.

From E. & S. LIVINGSTONE:

*Food and Physical Fitness.* By E. W. H. CRUICKSHANK, M.D., D.Sc., M.R.C.P., F.R.S.E. Price 5s.

From OXFORD UNIVERSITY PRESS:

*Actinomycosis.* By Zachary Cope, B.A., M.D., M.S. (Lond.), F.R.C.S. (Eng.). Price 15s.

*Collected Papers on Tuberculosis.* By Sir ROBERT W. PHILIP, M.A., M.D., LL.D., F.R.C.P., Hon. F.R.C.S.E., F.R.S.E. Price 21s.

*The Physiology of the Kidney.* HOMER W. SMITH, A.B., Sc.D., M.S. (Hon.). Price 15s.

*A Survey of Chronic Rheumatic Diseases.* Price 18s.

From JOHN WRIGHT & SONS LTD.:

*Emergency Surgery.* By HAMILTON BAILEY, F.R.C.S. (Eng.). Ed. 3. Price 50s.

*British Journal of Surgery.* Vol. XXV. No. 99 (January, 1938). Price 12s. 6d.

### Local Medical Notes

The twenty-seventh Long Fox Memorial Lecture will be delivered on Tuesday, 15th November, 1938, by Professor A. M. Tyndall, D.Sc., F.R.S. Subject: "Air as a Conductor of Electricity."

### EXAMINATION RESULTS.

**University of Bristol.**—Students of the University have recently passed the following examinations:—

M.B., Ch.B.—*Second Examination* (*Section I*). *Pass:* R. F. Brooks, J. H. Challenger, Doris E. Counsell, J. Deighton, Moragh J. Dickson, Mary D. Dixon, Eileen M. Herbert, Elisabeth Hodson, J. C. O. Iwenofu, A. G. H. Mitchinson, J. L. Pardoe, Margaret Savile, N. Slade, I. F. R. Sutherland, W. H. E. Turnbull, G. H. Valentine, Jean Wallace, Isabel M. M. Wilson.

**University of Cambridge.**

M.B., B.Chir.—*Third Examination* (*Part I*). *Pass:* C. H. Bartlett.

**Conjoint Board.**—L.R.C.P., M.R.C.S.—*Final Examination. Pass: In Medicine:* J. H. Bulleid,* P. W. J. Parkes. *In Surgery:* Myrtle M. Hutchins, Constance A. Mallett, Mabel L. Glenny. *In Midwifery:* Barbara Cunningham, Dorothy E. M. Dickinson.

L.D.S., R.C.S.—*First Professional Examination. Pass: In Dental Mechanics and Dental Metallurgy:* R. J. Gething.

* Qualified.

---

**Colston Research Society.**—The Thirty-fourth Annual Dinner of the Colston Research Society will be held in the Reception Room of the University of Bristol on Wednesday, 18th May next, at 7 o'clock for 7.30 p.m., under the Presidency of Mr. R. J. Sinclair. The guests of the evening are to be Professor D. M. S. Watson, F.R.S., Professor of Zoology and Comparative Anatomy, University of London, and Mr. Bernard Darwin, C.B.E.

For further particulars apply to the Hon. Secretary of the Colston Research Society, 12 Small Street, Bristol 1.

---

**Colston Medical Research Fellowships.**—The Council of the Colston Research Society, Bristol, offers one or more Fellowships for 1938–39 of not less than £100 per annum for Research work primarily in Clinical subjects, though other medical subjects are not excluded. The work must be carried out in the University of Bristol or in any of the Clinical Institutions associated with the University.

Awards will be made in June or July, 1938. Applications should be forwarded forthwith to the Hon. Secretary of the Colston Research Society, 12 Small Street, Bristol 1.

### Bristol Medico-Chirurgical Society.

### President.

C. A. MOORE, M.B., M.S. (Lond.), F.R.C.S.

### President-Elect.

L. A. MOORE, M.B., Ch.B. Brist.

### Committee.

*Ex-Officio* { J. A. NIXON, *C.M.G.*, B.A., M.D. Cantab., F.R.C.P. (Editor *Journal*).

(Vacant), (Hon. Librarian).

*Elected.*

R. C. CLARKE, *O.B.E.*, M.B., Ch.B.Brist. (Ex-President).
H. H. CARLETON, M.A., M.D., B.Ch.Oxon.
C. CORFIELD, M.D. Durh.
A. L. TAYLOR, M.D. Lond.
P. S. MARTIN, *M.C.*
T. MILLING, M.B., Ch.B. Belf.
W. V. WOOD, *M.C.*, B.A. Cantab.
H. L. SHEPHERD, M.B., Ch.M. Brist.
W. A. JACKMAN, F.R.C.S.

### Honorary Secretary.

R. V. COOKE, Ch.M.

### Honorary Treasurer.

A. E. ILES, *O.B.E.*, M.B., B.S. Lond., F.R.C.S.

### University Library Sub-Committee.

A. R. SHORT, B.Sc., M.D. Lond., F.R.C.S.
C. BRUCE PERRY, M.D. Brist., F.R.C.P.
J. H. DREW SMYTHE, *M.C.*, M.S., M.D. Lond., F.R.C.S., F.C.O.G.

Medical Practitioners wishing to join the Society are requested to communicate with the Hon. Secretary, Mr. R. V. Cooke, Failand Lodge, Guthrie Road, Clifton. The Annual Subscription is £1 1s., payable to Hon. Treasurer.

---

*Extracts from Journal By-laws.*

4.—The *Journal* shall be delivered free to Subscribers and also to Members of the Society whose subscriptions are not in arrear.

6.—The Annual Subscription to the *Journal* is 10/6, payable to Hon. Treasurer.

---

Communications intended for insertion in the *Journal* should be sent to the Editor, Dr. J. A. Nixon, 7 Lansdown Place, Clifton, Bristol.

Books for Review and Exchange Journals should be sent to the Assistant-Editor, *Bristol Medico-Chirurgical Journal,* Medical Library, The University, Bristol. Reviews should be returned with the books to the Assistant Editor at 65 Pembroke Road, Clifton, Bristol 8.

Communications referring to the delivery of the *Journal* should be sent to the Editorial Secretary, A. L. Flemming, 48 Pembroke Road, Clifton, Bristol.

Cases for Binding Vols. I—LIV are now ready, and may be obtained price 1/9 each (postage extra), upon application to J. W. Arrowsmith Ltd., Quay Street, Bristol; or the *Journal* will be bound, including Case, for 7/- per volume. The inclusive index for Volumes I—L may be obtained, price 3/- (postage extra).

### Bristol Medico-Chirurgical Society.

### PAST PRESIDENTS.

1874—75. FREDERICK BRITTAN, M.D., B.S. Dub.
1875—76. ROBERT WILLIAM COE.
1876—77. SAMUEL MARTYN, M.D. Ed.
1877—78. AUGUSTIN PRICHARD, M.D. Berlin.
1878—79. HENRY EDWARD FRIPP, M.D. Würzburg.
1879—80. WILLIAM MICHELL CLARKE.
1880—81. JOSEPH GRIFFITHS SWAYNE, M.D. Lond.
1881—82. EDWARD LONG FOX, M.D. Oxon.
1882—83. JAMES GEORGE DAVEY, M.D. St. And.
1883—84. WILLIAM JOHNSTONE FYFFE, M.D., B.A. Dub.
1884—85. GEORGE FORSTER BURDER, M.D. Aberd.
1885—86. WILLIAM HENRY SPENCER, M.D., M.A. Cantab.
1886—87. FRANCIS POOLE LANSDOWN.
1887—88. LEMUEL MATTHEWS GRIFFITHS.
1888—89. ROBERT SHINGLETON SMITH, M.D., B.Sc. Lond.
1889—90. NELSON CONGREVE DOBSON, Ch.M. Brist.
1890—91. SAMUEL HENRY SWAYNE.
1891—92. FRANCIS RICHARDSON CROSS, M.B. Lond., LL.D. Brist.
1892—93. EDWARD MARKHAM SKERRITT, M.D., B.S., B.A. Lond.
1893—94. JAMES GREIG SMITH, M.B., C.M., M.A. Aberd., F.R.S.E.
1894—95. ALFRED JAMES HARRISON, M.B. Lond.
1895—96. ARTHUR WILLIAM PRICHARD.
1896—97. ALFRED EDWARD AUST LAWRENCE, M.D., C.M. Aberd.
1897—98. JOHN EDWARD SHAW, M.B., C.M. Ed.
1898—99. ROBERT ROXBURGH, M.B. Ed.
1899—00. WILLIAM HENRY HARSANT.
1900—01. DAVID SAMUEL DAVIES, M.D. Lond.
1901—02. BARCLAY JOSIAH BARON, M.B., C.M. Ed.
1902—03. GEORGE MUNRO SMITH, M.D. Brist.
1903—04. JAMES PAUL BUSH, C.M.G., C.B.E., Ch.M. Brist
1904—05. REGINALD EAGER, M.D. Lond.
1905—06. JOHN DACRE.
1906—07. JAMES TAYLOR.
1907—08. HENRY WALDO, M.D., C.M. Aberd.
1908—09. JOHN MICHELL CLARKE, M.D., M.A. Cantab.
1909—10. JAMES SWAIN, C.B., C.B.E., M.D., M.S. Lond.
1910—11. BERTRAM MITFORD HERON ROGERS, M.D., B.Ch., B.A. Oxon.
1911—12. CHARLES ALEXANDER MORTON.
1912—13. WALTER CARLESS SWAYNE, M.D., B.S. Lond. and Brist.
1913—14. PATRICK WATSON-WILLIAMS, M.D. Lond., M.D. (Hon. Causa) Brist.
1914—15. WILLIAM HARRY CHRISTOPHER NEWNHAM, M.A., M.B. Cantab.
1915—16. GEORGE PARKER, M.A., M.D. Cantab., LL.D. Brist.
1916—17. FRANCIS HENRY EDGEWORTH, M.A., M.D. Cantab., D.Sc. Lond.
1917—18. FREDERICK LACE.
1918—19. ROBERT GUTHRIE POOLE LANSDOWN, M.D., B.S. Durh.
1919—20. I. WALKER HALL, M.D., Ch.B. Vict.
1920—21. LEOPOLD ERNEST VALENTINE EVERY-CLAYTON, M.D. Lond.
1921—22. CYRIL HUTCHINSON WALKER, B.A., M.B. Cantab.
1922—23. JOHN ALEXANDER NIXON, C.M.G., B.A., M.D. Cantab.
1923—24. JOHN LACY FIRTH, M.D., M.S. Lond.
1924—25. JOHN ODERY SYMES, M.D. Lond.
1925—26. THOMAS CARWARDINE, M.S. Lond.
1926—27. ARTHUR LAUNCELOT FLEMMING, M.B., Ch.B. Brist.
1927—28. WALTER KENNETH WILLS, M.A., M.B., B.Ch. Cantab.
1928—29. HENRY LAWRENCE ORMEROD, M.D., B.Ch. R.U.I.
1929—30. CHARLES FERRIER WALTERS.
1930—31. DAVID CHARLES RAYNER, Ch.M. Brist.
1931—32. JOHN ROGER CHARLES, M.A., M.D. Cantab.
1932—33. ERNEST WILLIAM HEY GROVES, M.D., M.S., D.Sc., B.Sc. Lond.
1933—34. HERBERT ELWIN HARRIS, B.A., M.B. Cantab.
1934—35. A. RENDLE SHORT, B.Sc., M.D. Lond.
1935—36. ARTHUR ERNEST ILES, O.B.E., M.B., B.S. Lond.
1936—37. RICHARD CHRISTOPHER CLARKE, O.B.E., M.B. Lond.

1938.

### LIST OF SUBSCRIBERS

TO THE

### Bristol Medico-Chirurgical Journal.

### HONORARY MEMBERS OF THE SOCIETY.

SWAIN, JAMES, *C.B.*, *C.B.E.*, M.D., M.S. Lond. .. .. .. Wyndham House, Easton-in-Gordano.
WATSON-WILLIAMS, P., M.D. Lond. 2 Rodney Place, Clifton, Bristol 8.

### SUBSCRIBERS.

*Those marked * are Members of the Society.*

*ABBOTT, Miss E. K., M.B., Ch.B. (Otago) .. X-Ray Department, Bristol General Hospital.
*ACKLAND, W. R., M.D.S. .. .. .. .. .. .. 5 Rodney Place, Clifton, Bristol 8.
*ADAMS, A. W., M.S. Lond. .. .. .. .. .. Rodney House, Clifton, Bristol 8.
ADAMS, J. B. .. .. .. .. .. .. .. .. .. Kent Lodge, Eastbourne.
*ADAMS, S.B., Ph.D., M.B., Ch.B. Brist. .. .. 19 Charlotte Street, Bristol 1.
*ALDWINCKLE, HELEN M., M.B., Ch.B. Brist. .. 1 Manor Road, Fishponds, Bristol.
*ALEXANDER, A. J. P., M.D. Belf. .. .. .. Winsley Sanatorium, near Bath.
*ALEXANDER, D. A., M.B., Ch.B. Vict. .. .. 112 Pembroke Road, Clifton, Bristol 8.
ALEXANDER, G. L., B.A., M.B., B.Ch. Cantab. West African Medical Service, Laura, via Accra, Gold Coast.
*ALFORD, A. F., M.B., Ch.B. Brist. .. .. .. ..
ALFORD, H. J., M.D. Lond. .. .. .. .. .. 4 Park Terrace, Taunton.
*ARMSTRONG, JEAN, M.B., Ch.B. Brist. .. .. Succoth, Duckmoor Road, Ashton Gate, Bristol 3.
ASHTON, L. P., M.B., Ch.B. Brist. .. .. .. Kapsowar, P.O. Eldoret, Kenya, East Africa.
*AUBREY, T., M.B. Lond. .. .. .. .. .. .. Bitton, near Bristol.

*BAIN, W. .. .. .. .. .. .. .. .. .. .. 1 Greville Road, Southville, Bristol 3.
*BAKER, LILY A., M.D., B.Ch., B.A.O., R.U.I. Clifton Down House, Beaufort Buildings, Clifton, Bristol 8.
*BARBER, M. C., M.B., Ch.B. Brist. .. .. .. Ingledene, High Street, Staple Hill, Bristol.
*BARBOUR, R. F., M.A. Cantab., M.B., Ch.B. 16 Mortimer Road, Clifton, Bristol.
*BARKER, G. H., M.D., B.Sc. Lond. .. .. .. 124 Redland Road, Bristol 6.
*BARTHOLOMEW, E. U. .. .. .. .. .. .. .. 12 Lansdown Place, Clifton, Bristol 8.
*BATES, R. M. .. .. .. .. .. .. .. .. .. Purdown House, Stapleton, Bristol 5.
BEATSON, D. H., M.B., Ch.B. Brist. .. .. 8 Richmond Park Road, Clifton, Bristol 8.
*BERGIN, F. G. .. .. .. .. .. .. .. .. .. 31 Oakfield Road, Clifton, Bristol 8.
*BERRY, R. J. A., Prof., M.D., C.M. Edin. .. Director of Medical Services, Stoke Park Colony, Bristol 5.
*BIRRELL, J. A., M.D., B.S. Lond. .. .. .. 36 Kellaway Avenue, Bristol 6.
*BLACHFORD, J. V., *C.B.E.*, M.D. Durh. .. .. Milverton House, Long Ashton, near Bristol.
BLACKETT, J. F., M.D., B.S. Lond. .. .. .. Hamsterley, Bloomfield Park, Bath.
BLETCHLY, G. PLAYNE, M.B. Lond. .. .. 35 Stratford Road, Summerton, Oxford.
*BODMAN, A. P., M.B., Ch.B. .. .. .. .. .. 11 Hurle Crescent, Clifton, Bristol 8.
*BODMAN, C. O., M.D. Durh. .. .. .. .. .. 17 Upper Belgrave Road, Clifton, Bristol 8.
*BODMAN, F. H., M.D., Ch.B. Brist. .. .. .. 2 Redland Court Road, Bristol 6.
BOLT, R. F. .. .. .. .. .. .. .. .. .. .. 70 Great Russell Street, London, W.C.1.
*BOULTON, B. J., M.B., Ch.B. Brist. .. .. Ham Green Hospital, Pill, near Bristol.
*BOWN, NAOMI J., M.B., Ch.B. Brist. .. .. Demeter, Wraxall, Somerset.
*BRADBEER, E. G., M.B., Ch.B. Brist. .. .. 13 Percival Road, Bristol 8.
BRASHER, C. W. J. .. .. .. .. .. .. .. .. 62 Queen Anne St., Cavendish Square, London, W.1.
*BRINCKMAN, WINIFRED P., M.B., Ch.B. Brist. 4 Oakland Road, Redland, Bristol 6.
*BRITTON, R. B., M.B., B.S. Lond. .. .. .. 33a Whiteladies Road, Clifton, Bristol 8.
*BROCKLEHURST, R. J., M.A., D.M. Oxon. .. The University, Bristol 8.
BROWN, J. E., M.B., Ch.B. Brist. .. .. .. Coulthurst Villa, Brives Road, Maraval, Trinidad, B.W.I.
*BURGES, R. .. .. .. .. .. .. .. .. .. .. Druid's Mead, Stoke Bishop, Bristol 9.

| | |
|---|---|
| *BURGESS, N. F. C., M.D. Cantab. .. .. .. | Hereford House, Clifton, Bristol 8. |
| *BUSH, G. B., M.B., Ch.B. Brist. .. .. .. | 132 Queen's Road, Clifton, Bristol 8. |
| *CAIRNS, F. J... .. .. .. .. .. .. .. .. | Devon House, Whitehall Road, Bristol 5. |
| *CAPPER, W. M., M.B., B.S. Lond... .. .. | Bristol Royal Infirmary, Bristol. |
| *CAREY, R. S., *O.B.E.*, B.A. Cantab. .. .. | 502 Bath Road, Brislington, Bristol 4. |
| *CARLETON, H. H., M.A., M.D., B.Ch. Oxon. | 1 Lansdown Road, Clifton, Bristol 8. |
| *CASSON, E., M.D., Ch.B. Brist. .. .. .. .. | Dorset House, Clifton Down, Bristol 8. |
| *CATES, H. J., M.D. Lond. .. .. .. .. .. | Northwoods House, Winterbourne, Bristol. |
| *CHAMBERS, E. R. .. .. .. .. .. .. .. .. | 9 Clifton Park, Clifton, Bristol 8. |
| *CHARLES, J. R., M.A., M.D. Cantab. .. .. | Litfield House, Clifton Down, Bristol 8. |
| *CHITTY, H., M.S. Lond. .. .. .. .. .. .. | 46 Pembroke Road, Clifton, Bristol 8. |
| *CHRISTOFFERSON, P. E., M.B., Ch.B. Brist. .. | 4 Victoria Square, Clifton, Bristol 8. |
| *CLAREMONT, L. E., M.D.S. Brist. .. .. .. | 5 Rodney Place, Clifton, Bristol 8. |
| *CLARK, DENNIS O. .. .. .. .. .. .. .. | 168 Milton Road, Bristol Road, Weston-super-Mare. |
| *CLARKE, R. C., *O.B.E.*, M.B., Ch.B. Brist. .. | 29 Victoria Square, Clifton, Bristol 8. |
| *CLUTTERBUCK, E. R., M.B., Ch.B. Brist. .. | 63 Walliscote Road, Weston-super-Mare. |
| *COCHRANE, J. H. .. .. .. .. .. .. .. | Royal Infirmary, Bristol. |
| *COLLINGWOOD, F. C., M.B., Ch.B. Brist. .. | Southmead Hospital. |
| CONNELLAN, P. S... .. .. .. .. .. .. .. | Marmion, Shortheath Road, Farnham, Surrey. |
| *COOKE, ELIZABETH M., M.D. Lond. .. .. | Failand Lodge, Guthrie Road, Clifton. |
| *COOKE, R. V., Ch.M. Brist. .. .. .. .. | Failand Lodge, Guthrie Road, Clifton. |
| *COOKSON, R. G. .. .. .. .. .. .. .. .. | 5 Worcester Road, Clifton, Bristol 8. |
| *COOMBS, C. F., B.Ch. Cantab. .. .. .. .. | 14 Southfield Road, Westbury-on-Trym, Bristol. |
| COOMBS, N. C. .. .. .. .. .. .. .. .. | Romaldkirk, Barnard Castle, Co. Durham. |
| *COOPER, W. R. .. .. .. .. .. .. .. .. | 13 Westfield Park, Redland, Bristol 6. |
| *CORFIELD, C., M.D. Durh. .. .. .. .. .. | 5 Kensington Place, Clifton, Bristol 8. |
| *CORNER, BERYL, M.D., B.S. Lond. .. .. .. | 3 Elton House, Rodney Place, Bristol 8. |
| *COSSHAM, W. L., M.B., Ch.B. Brist. .. .. | 3 Claremont Road, Bishopstcn, Bristol 7. |
| *COURTNEY, R. M., M.B., Ch.B. Glasg. .. .. | 487 Gloucester Road, Bristol 7. |
| *COXON, BEATRICE .. .. .. .. .. .. .. | 16 Richmond Hill, Clifton, Bristol 8. |
| *CRAIG, ANNE A., M.B., B.S. Lond. .. .. .. | 18 Vyvyan Terrace, Clifton, Bristol 8. |
| *CROOK, B. A., M.B., Ch.B. Brist. .. .. .. | The Laurels, Timsbury, near Bath. |
| *CROSSMAN, F. W., M.B. Durh. .. .. .. .. | White's Hill, Hambrook, near Bristol. |
| *DATTA, S. S., M.D. Brist. .. .. .. .. .. | Snowdon House, Fishponds, Bristol. |
| *DAVIE, T. B., M.D. Liverpool .. .. .. .. | Canynge Hall, Clifton, Bristol 8. |
| *DAVIES, E. D. D. .. .. .. .. .. .. .. | Standish House, Stonehouse, Glos |
| *DAWSON, W. R., *O.B.E.*, M.D. Dub. .. .. | 18 Brock Street, Bath. |
| *DELANEY, N., M.B., Ch.B. Manch. .. .. .. | The Meade, Bishopsworth, Somerset. |
| *DELICATI, J. L. .. .. .. .. .. .. .. | Royal Mineral Water Hospital, Bath. |
| DEVINE, H., *O.B.E.*, M.D. Lond. .. .. .. | Holloway Sanatorium, Virginia Water. |
| *DICK, A., M.B., Ch.B. Glasg. .. .. .. .. | Aldersyde, Birstall, Leeds. |
| *DICKSON, W. A., M.D. St. And. .. .. .. | Standish House, Stonehouse, Glos. |
| *DISNEY, M., M.D. Lond. .. .. .. .. .. | Bristol Royal Infirmary. |
| *DIX, C. .. .. .. .. .. .. .. .. .. .. | 14 Mortimer Road, Clifton, Bristol 8. |
| *DIXON, HELEN M., M.B., Ch.B. Brist. .. .. | 7 Mendip Gardens, Upper Wellsway, Bath. |
| *DIXON, T. B. .. .. .. .. .. .. .. .. | 11 Pembroke Road, Clifton, Bristol 8. |
| *DUERDEN, J. R., M.B., Ch.B. Brist. .. .. | 4 Cecil Road, Bristol 8. |
| *DUNLEVY, A. A., M.B., B.Ch. N.U.I. .. .. | 23 Pembroke Road, Clifton, Bristol. |
| *DUTTON, HILDA R. .. .. .. .. .. .. .. | 405 Stapleton Road, Bristol 5. |
| *DYER, W. P. .. .. .. .. .. .. .. .. | West View, Westbury-on-Trym, Bristol. |
| *EGLINTON, J. H. C. .. .. .. .. .. .. .. | Street, Somerset. |
| ELLIOTTS & AUSTRALIAN DRUG LTD. .. .. | 29 O'Connell Street, Sydney, N.S.W., Australia. |
| ELLIOTT, F. P., M.B., C.M. Aberd. .. .. .. | 113 Grove Road, Walthamstow, London, E.17. |
| *ELLIOTT, W. H. A., M.B., B.S. Lond. .. .. | 116 Pembroke Road, Clifton, Bristol 8. |
| *ELLIS, W. T. .. .. .. .. .. .. .. .. | Tregenna House, Shirehampton. |
| *ESKELL, NEIL P. .. .. .. .. .. .. .. | 167 Wells Road, Bristol. |

| | |
|---|---|
| *EVANS, G. M., M.B., Ch.B. Brist. .. .. .. | 117 Ashley Road, Bristol. |
| *EYRE-BROOK, A. L., M.B., M.S. Lond. .. .. | The Cottage, Portishead. |
| *FALLON, Col. J. .. .. .. .. .. .. .. .. | 35 Henleaze Gardens, Henleaze, Bristol. |
| *FARAKER, E. C. .. .. .. .. .. .. .. .. | 85 Coldharbour Road, Westbury Park, Bristol 6. |
| *FAULL, J. L. .. .. .. .. .. .. .. .. | 61 Cotham Brow, Bristol 6. |
| *FAWN, G. F. .. .. .. .. .. .. .. .. | 6 Oakfield Road, Clifton, Bristol 8. |
| *FELLS, A., M.B., C.M. Edin. .. .. .. .. | 19 Alexandra Road, Clifton, Bristol 8. |
| *FELLS, G. R., M.B., Ch.B. Brist. .. .. .. | 19 Southmead Road, Bristol. |
| *FELLS, R. R., M.B., B.S. Lond. .. .. .. | 17 Mortimer Road, Clifton, Bristol 8. |
| *FENELEY, G. L., M.B., Ch.B. Brist. .. .. | Englewood, Falcondale Road, Westbury-on-Trym. |
| *FINZEL, H. F. M., M.D. Brist. .. .. .. .. | Nare House, Birchwood Road, St. Anne's Park, Bristol 4. |
| *FIRTH, J. L., M.D., M.S. Lond. .. .. .. | 8 Victoria Square, Clifton, Bristol 8. |
| *FISHER, A. C., M.D. Brist. .. .. .. .. | Roan Antelope Mine, N. Rhodesia. |
| *FITZGIBBON, G. M., M.D. Lond. .. .. .. | 52 Pembroke Road, Clifton, Bristol 8. |
| *FITZGIBBON, Mrs. L. P., M.B., B.S. Lond. .. | 52 Pembroke Road, Clifton, Bristol 8. |
| *FLEMMING, A. L., M.B., Ch.B. Brist. .. .. | 48 Pembroke Road, Clifton, Bristol 8. |
| *FORRESTER-BROWN, MAUD, M.D., M.S. Lond. | 22 Combe Park, Bath. |
| *FOSS, G. L., M.B., B.Ch. Cantab. .. .. .. | Cloud's Hill House, St. George, Bristol 5. |
| *FOX, F. E., B.A. Cantab. .. .. .. .. .. | Brislington House, Brislington, Bristol 4. |
| *FRASER, A. D., M.A., M.B., Ch.B. Glasg. .. | 4 Alexandra Road, Clifton, Bristol 8. |
| *FRASER, D., M.B., Ch.B. Edin. .. .. .. | Westgate Dursley, Glos. |
| *FULLER, H. L. .. .. .. .. .. .. .. .. | 9 Gay Street, Bath. |
| *FURNIVAL, Col. C. H. .. .. .. .. .. .. | "Yatton," Wellington Hill, Horfield, Bristol 7. |
| *GARDEN, R. R., M.A., M.B., B.Ch. Aberd. .. | 9 Harley Place, Clifton, Bristol 8. |
| GARLAND, E. B., M.B., C.M. Edin. .. .. .. | 114a Hampton Road, Redland, Bristol 6. |
| *GEE, C. A. H., M.B. Ch.B. Brist. .. .. .. | Wansdyke, Portbury, near Bristol. |
| *GERRISH, NORMAN, M.B., Ch.B. Brist. .. .. | 330 Church Road, St. George, Bristol. |
| GIBBS, J. R. .. .. .. .. .. .. .. .. | 48 Codrington Road, Bishopston, Bristol 7. |
| *GLENNY, E. T., M.B., B.S. Lond. .. .. .. | 102 Pembroke Road, Clifton, Bristol 8. |
| *GORDON, R. G., M.D., B.Sc. Edin. .. .. .. | 2 Catharine Place, Bath. |
| *GORHAM, A. P., M.B., Ch.B. Brist. .. .. .. | 53 Westbury Road, Westbury-on-Trym. |
| *GORNALL, W. A., M.D. Brist. .. .. .. .. | Ulvik, Nailsea, near Bristol. |
| *GRAY, J. D. A., M.B., Ch.B., B.Sc. Edin. .. | 212 Redland Road, Bristol 6. |
| *GREEN, S. B., M.B. Lond. .. .. .. .. .. | The Black Wicket, Westbury-on-Trym. |
| *GREENLAW, MADGE E., M.B., Ch.B. Brist. .. | 194 Redland Road, Bristol 6. |
| GRIFFITHS, CORNELIUS A. .. .. .. .. .. | 35 Newport Road, Cardiff. |
| *GROVES, E. W. HEY, M.D., M.S., D.Sc. Lond. .. | 25 Victoria Square, Clifton, Bristol 8. |
| *GUIRDHAM, A., M.A., B.M., B.Ch. Oxon. .. | Bailbrooke House, Bath. |
| *HALL, E. GEORGE, M.B. Lond. .. .. .. .. | 42 Apsley Road, Clifton, Bristol 8. |
| *HAM, C. I. M.B., Ch.B. Brist. .. .. .. .. | Frenchay Park Sanatorium, Frenchay, near Bristol. |
| *HARRIS, H. ELWIN, M.A., M.B. Cantab. .. | The Mount, Halse, Taunton. |
| *HARRIS, H. E., M.A., M.B., B.Ch. Cantab. .. | 13 Lansdown Place, Clifton, Bristol 8. |
| HARRISON, C. C. .. .. .. .. .. .. .. .. | 44 High Street, Keynsham. |
| *HARTLEY, G., M.B., B.S. Lond. .. .. .. | 2 Harley Place, Clifton, Bristol 8. |
| *HAWKINS, E. J. .. .. .. .. .. .. .. .. | 27 Westbury Road, Westbury-on-Trym |
| *HEALES, J. N. .. .. .. .. .. .. .. .. | Holmcroft, Street, Somerset. |
| *HECTOR, F., M.D. Lond. .. .. .. .. .. | 1 Victoria Square, Clifton, Bristol 8. |
| *HEMPHILL, R., M.B., B.Ch. Dub. .. .. .. | Fishponds Mental Hospital, Bristol. |
| *HENDERSON, JAMES, M.B., Ch.B. Aberd. .. | The City Sanatorium, Yardley Road, Birmingham. |
| *HERAPATH, C. E. K., *M.C.*, M.D. Lond. .. | Ormlie, Clifton, Bristol 8. |
| *HERON, GORDON, M.B., Ch.B. Brist. .. .. | 34 Gloucester Road, Bristol 7. |
| *HERON, DORIS, M.B., Ch.B. Brist. .. .. .. | 34 Gloucester Road, Bristol 7. |
| *HILL, WINIFRED, M.B., Ch.B. Brist. . .. | 49 Tudor Gardens, West Acton, W. 3. |
| *HOLLAND, W. A. L. .. .. .. .. .. .. .. | 10 Brunswick Square, Bristol. |
| *HOOKER, J. A., M.B., Ch.B. Brist. .. .. .. | 16 St. Edyth's Road, Sea Mills 9. |
| *HOSKEN, J. G. F... .. .. .. .. .. .. .. | Uley, Glos. |
| HOSPITAL, BRISTOL GENERAL (*Secretary*, T. W. GREGG). | |
| *HOUGHTON, LYDIA M., M.B., B.S. Lond. .. | 74 Church Road, Redfield, Bristol 5. |

H

| | |
|---|---|
| *HOWELL, H. | Kincraig, Beach Road, Weston-super-Mare. |
| *HUGHES, F. W. TERRELL, M.B., Ch.B. Brist. | Chew Magna, Somerset. |
| *HUGHES, MARGUERITE G., M.B., Ch.B. Brist. | 12 Upper Belgrave Road, Clifton, Bristol 8. |
| *HUNTER, F. S. | c/o Messrs. J. Wright & Sons Ltd., Publishers, Bristol 1. |
| *HUTCHINSON, BRENDA | 23 The Circus, Bath. |
| *HUTCHINSON, C. A. | 23 The Circus, Bath. |
| *HYATT, ANNIE W., M.B., B.S. Lond. | Merrymead, Shepton Mallet, Som. |
| *ILES, A. E., *O.B.E.*, M.B., B.S. Lond. | 87 Pembroke Road, Clifton, Bristol 8. |
| INFIRMARY, BRISTOL ROYAL (*Secretary*, E. C. SMITH). | |
| INGLE, C. DURBAN | Somerton, Somerset. |
| *JACKMAN, W. A., M.B., Ch.B. Brist. | 15 Mortimer Road, Clifton, Bristol 8. |
| *JAMES, APRIL D., M.B., Ch.B. Brist. | |
| *JAMES, J. A, M.D. Lond. | Litfield House, Clifton Down, Bristol 8. |
| *JAMES, J. A. Senr. | 43 Nevil Road, Bishopston, Bristol. |
| *JENKINS, F. G., M.B., Ch.B. Brist. | 51 Redcliff Hill, Bristol 1. |
| *JENKINS, R. D. | The Copper Beeches, Almondsbury, near Bristol. |
| *JENKINS, P. K., M.B., Ch.B. | 29 Downs Park West, Bristol 6. |
| *JOHNSON, R. G., M.B. Lond. | 119 Redland Road, Bristol 6. |
| *JOLL, C. A., M.D., M.S. Lond. | 64 Harley Street, London, W.1. |
| *JORDAN, L. R., M.B., Ch.B. Brist. | National Temperance Hospital, Hampstead Road, N.W.1. |
| *JOSCELYNE, P. C., M.B., Ch.B. Brist. | 70 Longmead Avenue, Bishopston, Bristol 7. |
| *KEMM, N. F., M.B., B.S. Lond. | 200 Redland Road, Durdham Park, Bristol, 6. |
| KING, E. F. | 2 Upper Wimpole Street, London, W.1. |
| *KINGSTON, S. H., M.B., Ch.B. Brist. | 3 Whiteladies Road, Clifton, Bristol 8. |
| *KYLE, H. G., M.A., M.D., B.Ch. Oxon. | 31 Westbury Road, Bristol. |
| *LACE, F | 5 Gay Street, Bath. |
| LALONDE, T. P., M.B., Ch.B. Brist. | Wykeham House, Romsey, Hants. |
| *LEES, E. LEONARD, M.D., C.M. Edin. | 22 Richmond Hill, Clifton, Bristol 8. |
| *LEGAT, R. EDDOWES, M.B., C.M. Edin. | Murray House, Staple Hill, Bristol. |
| *LEWIS, F. J., M.B., Ch.B. Brist. | The Royal Infirmary, Bristol. |
| *LING, T. M., M.A., B.M., B.Ch. | "St. Budoc," Elmlea Avenue, Stoke Bishop, Bristol. |
| *LINTON, MARION S., M.B. Lond. | 1 Brecon Road, Henleaze, Bristol. |
| *LISTER, A. E. J., M.B., B.S. Lond. | 86 Pembroke Road, Clifton, Bristol 8. |
| *LISTER, L. MARGARET | 12 All Saints Road, Clifton, Bristol 8. |
| LOGAN, H. B. | Elmwood, Aberdeen Road, Cotham, Bristol 6. |
| *LOXTON, S. D., M.B., Ch.B. | 17 Oakfield Grove, Clifton, Bristol 8. |
| *LUCAS, J. E. | 48 Coronation Road, Bristol 3. |
| *LUCAS, J. J. S., M.D. Lond. | 186 Wells Road, Totterdown, Bristol 4. |
| *LUCAS, J. V. | 186 Wells Road, Knowle, Bristol 4. |
| *LUCAS R. P., M.B., Ch.B. Brist. | 15 Edgecumbe Road, Redland Bristol 6. |
| *McCREA, PHILLIP, M.D., B.Ch. Dub. | 56 Kellaway Avenue, Bristol 6. |
| *McKENZIE, W. G., *M.C.* | 10 Woodland Road, Surbiton. |
| *MACLACHLAN, A. M., M.B., Ch.B. Glasg. | 65 Redcliff Hill, Bristol 1. |
| *McLANNAHAN, J. G. | The Mount, Stonehouse. |
| *MACLAREN, CATHERINE J., M.B., Ch.B. (Edin.) | Hereford House, Clifton Down. |
| MACLEAN, SIR EWEN J., M.D., C.M. Edin. | 12 Park Place, Cardiff. |
| *MACLEOD, DONALD, M.B., C.M. Edin. | 84 Redland Road, Redland, Bristol 6. |
| *MACLEOD, G., M.A. Glas., M.D., M.B. | Wargrave, Clevedon, Somerset. |
| *MARSHALL, A. H., M.B., Ch.B. Brist. | Odelos, Canford Lane, Bristol. |
| MARSHALL, A. L., M.B., B.A. Cantab. | Laxfield, Woodbridge, Suffolk. |
| *MARTIN, J. E., M.B., Ch.B. | 32 Whiteladies Road, Clifton. |
| *MARTIN, J. J. B., M.A., M.D. Edin. | Bristol Mental Hospital. |
| *MARTIN, P. S., *M.C.* | Victoria Quadrant, Weston-super-Mare. |
| *MAYES, F. J. A. | 23 Pembroke Road, Clifton, Bristol 8. |
| *MEADOWS, G. | W. D. & H. O. Wills, No. 1 Factory, East Street, Bedminster, Bristol 3. |
| MIALL, C. L'O | Elm Hayes, Paulton, near Bristol. |

| | |
|---|---|
| *Milling, T., M.B., Ch.B. Belf. | 1 Vicarage Road, Southville, Bristol 3. |
| *Milner, A., M.B., B.Ch. Dub. | 61 Cotham Brow, Bristol 6. |
| *Moore, C. A., M.B., M.S. Lond. | 56 St. Paul's Road, Clifton, Bristol 8. |
| *Moore, L. A., M.B., Ch.B. Brist. | Hillsborough, Cotham Park, Bristol 6. |
| *Moore, R. H., M.B., Ch.B. Brist. | White Cross Court, Wells Road, Bristol 4. |
| *Morgans, C. C., M.B., Ch.B. Cantab. | 65 Lower Redland Road, Bristol 6. |
| *Morris, L. N., M.B., Ch.B. Brist. | 13 Belgrave Road, Clifton, Bristol 8. |
| *Mowat, F. D. | 33 Canynge Road, Clifton, Bristol 8. |
| *Mundy, G. S., M.B., B.Ch. Brist. | Homewood, Coombe Dingle, Bristol. |
| *Myles, G. T. | 7 Apsley Road, Clifton, Bristol 8. |
| *Myles, Q. St. L., M.A., B.M., B.Ch. Oxon. | 7 Apsley Road, Clifton, Bristol 8. |
| *Newlands, H. J. | Moray House, Fishponds, Bristol. |
| *Nicholson-Lailey, J. R. | 8 Mount Terrace, Taunton. |
| *Nixon, Doreen, M.B., B.S. Lond. | 7 Lansdown Place, Clifton, Bristol 8. |
| *Nixon, J. A., *C.M.G.*, M.D. Cantab. | 7 Lansdown Place, Clifton, Bristol 8. |
| *Noble, J. I., M.B., Ch.B. Liverp. | 102 Pembroke Road, Clifton, Bristol 8. |
| *Norman, R. M., M.D. Brist. | 25 Victoria Square, Clifton, Bristol 8. |
| *Nott, Winifred, M.B., Ch.B. Brist. | Fassiefern, 8 Rylestone Grove, Stoke Bishop, Bristol 9. |
| *O'Donohoe, Monica A., M.B., Ch.B. | 19 Westfield Park, Redland, Bristol 6. |
| *Oliver, L. C. | The General Hospital, Bristol. |
| *Ormerod, G. L. M.A., M.B., B.Ch. Cantab | 2 Henleaze Road, Westbury-on-Trym, Bristol. |
| *Orr-Ewing, H. J., *M.C.*, M.D. Lond. | 1 Beaufort Buildings, Clifton, Bristol 8. |
| *Palin, A. G. | 23 Pembroke Road, Bristol 8. |
| *Parkes, J. A., M.B., Ch.B. Liverp. | 8 South Road, Redland, Bristol 6. |
| Parkinson, Lt.-Col. G. S., *D.S.O.*, R.A.M.C. | London School of Hygiene and Tropical Medicine, Keppel Street, London, W.C. 1. |
| *Parry, R. H., M.B., B.S. Lond. | Public Health Offices, 40 Prince Street, Bristol 1. |
| Parsons, Sir J. H., M.B., B.S., B.Sc. Lond. | 54 Queen Anne Street, London, W. |
| *Paul, R. G. | 23 Pembroke Road, Bristol 8. |
| *Perry, B., M.D., Ch.B. Brist. | 4 Richmond Park Road, Clifton, Bristol 8. |
| *Philipp, E., M.B., Ch.B. | 521 Filton Avenue, Bristol 7. |
| *Phillips, L. B., M.B., Ch.B. Brist. | Holdenhurst, Filton, Bristol. |
| *Phillips, P., M.B., Ch.B. Brist. | Southmead Hospital, Bristol. |
| *Pineo, E. D. | Richmond House, Langford, Som. |
| *Pocock, J. A., M.A., M.B., B.Ch. (Cantab.) | General Hospital, Bristol. |
| *Pollard, J., M.B., B.Ch., B.A.O., N.U.I | 88 Coronation Road, Bristol 3. |
| Porter, C. P. | 28 Mill Street, Kidderminster. |
| *Potter, Mabel F., M.B., Ch.B. Brist. | 23 Pembroke Road, Clifton, Bristol 8. |
| *Powell, H. F., M.D. Brux. | Ellenborough Park, Weston-super-Mare. |
| *Price, C. H. G., M.B., Ch.B. Brist. | 6 Lansdown Place, Bristol 8. |
| *Price, N. L., M.B., Ch.B. Brist. | 9 Royal Park, Clifton, Bristol 8. |
| *Pridie, K. H., M.B., B.S. Lond. | 58 St. John's Road, Clifton, Bristol 8. |
| *Pringle, Miss J. L., M.B., Ch.B. Edin. | 115 Lower Oldfield Park, Bath. |
| *Prowse, D. C., M.B., Ch.B. Brist. | Wigmore House, Thornbury, near Bristol. |
| *Pyke, H. D., M.B., Ch.B. Brist. | 88 Redland Road, Bristol 6. |
| *Rankin, P. C., M.B., Ch.B. Glasg. | Lawrence Hill House, Bristol. |
| *Rayner, D. C., Ch.M. Brist. | 9 Lansdown Place, Clifton, Bristol 8. |
| *Redman, Ethel, M.B., Ch.B. | 55 Bridgwater Road, Bedminster Down, Bristol 3. |
| *Roberts, J. A. F., M.A. Cantab., M.B., Ch.B., D.Sc. Edin. | Stoke Park Colony, Bristol. |
| *Robertson, D., M.B., Ch.B. Edin. | 2 Knowle Road, Knowle, Bristol 4. |
| *Robertson, L. G., M.B., Ch.B. | 162 Redland Road, Bristol 6. |
| *Rogers, H., M.D. Brist. | 91 Old Park Ridings, Grange Park, N.21. |
| *Rolfe, R., B.A., M.B., B.C. Cantab. | Park House, St. Andrew's Road, Avonmouth. |
| *Rowley, A. T. | 13 Walliscote Road, Weston-super-Mare. |
| *Rudolf, G. R. A. de M. | 5 Pembroke Road, Clifton, Bristol 8. |
| *Ryan, P. B., M.B., Ch.B. | General Hospital, Bristol. |
| *Scarff, G., M.B., Ch.B. Edin. | 83 Pembroke Road, Clifton, Bristol 8. |
| *Shaw, J. E., M.B., C.M. Edin. | 23 Caledonia Place, Clifton, Bristol 8. |

| | |
|---|---|
| *SHEPHERD, H. L., M.B., Ch.M. Brist. .. .. | 79 Pembroke Road, Clifton, Bristol 8. |
| *SHORT, A. R., B.Sc., M.D., B.S. Lond. .. .. | 69 Pembroke Road, Clifton, Bristol 8. |
| *SILBERSTEIN, ANNA H., M.B., Ch.B. Brist. .. | The Children's Hospital, Bristol. |
| *SMITH, T. W., M.D. Lond.. .. .. .. .. .. | 26 The Circus, Bath. |
| *SMYTHE, H. J. D., *M.C.*, M.D., M.S. Lond. .. | 15 Eaton Crescent, Clifton, Bristol 8. |
| *SNAWDON, J. W. E., M.B., Ch.B., B.D.S. .. | Royal Infirmary, Bristol. |
| *SOLTAU, H. K. V., M.D. Lond. .. .. .. | 19 The Avenue, Clifton, Bristol 8. |
| *SOMERS, MARY A. E. .. .. .. .. .. .. .. | 2 Ashcombe Road, Weston-super-Mare. |
| *SPOOR, A., M.B., B.Ch. Cantab. .. .. .. | The Hydro, Bristol 1. |
| *STANILAND, M. F... .. .. .. .. .. .. .. | 255 Stapleton Road, Bristol 5. |
| *STATHAM, R. S. S., *O.B.E.* M.D., M.Ch. Brist. | Cheddar, Som. |
| *STEVEN, G. D., M.B., Ch.B. Edin., D.P.H. .. | 6 Upper Church Street, Bath. |
| *STONE, DORIS M., M.D., B.S. Lond. .. .. | British Postgraduate School, Ducane Road Shepherds Bush, W.11. |
| *STROVER, H. W. M., *O.B.E.*, M.B., Ch.B. Aberd. | 12 Gloucester Road, Bishopston, Bristol 7. |
| *STRUTHERS, A. J., M.B., Ch.B. Glasg. .. .. | 100 Church Road, Redfield, Bristol 5. |
| *STRUTHERS, GEO., M.B., Ch.B. (Glasgow) .. | Southmead Hospital, Bristol. |
| *SUTTON, G. E. F., M.D. Lond. .. .. .. .. | 1 Pembroke Road, Clifton, Bristol 8. |
| *SYMES, J. O., M.D. Lond. .. .. .. .. .. | 6 Pembroke Vale, Clifton, Bristol 8. |
| *TASKER, D. G. C., M.B., M.S. Lond. .. .. | 9 College Fields, Clifton, Bristol 8. |
| *TAYLOR, A. L., M.D. Lond. .. .. .. .. | The General Hospital, Bristol 1. |
| TAYLOR, A. L. .. .. .. .. .. .. .. .. .. | The Royal Infirmary, Bristol 1. |
| *TAYLOR, CONSTANCE L., M.B., Ch.B. Edin. .. | Queen Charlotte's Hospital, Marylebone Road, N.W.1. |
| *TAYLOR, H. N., M.A. Cantab., M.D. Dub. .. | Rosetta, Stow Park Circle, Newport, Mon. |
| *TEMPLE, G. H., M.B., C.M. Edin. .. .. .. | Ailanthus, Weston-super-Mare. |
| THIN, J... .. .. .. .. .. .. .. .. .. .. | 54 and 55 South Bridge, Edinburgh. |
| *THOMAS J. D., M.B. Cantab. .. .. .. .. | Dan-y-Graig, Cheyne Road, Stoke Bishop, Bristol 9. |
| *TODD, A. T., *O.B.E.*, M.D. Edin. .. .. .. | Royal Park House, Clifton, Bristol 8. |
| TRIPP, DOROTHY M. H. .. .. .. .. .. .. | Mission Hospital, Karim Nagar, Nizam's Dominions, South India. |
| *TRIST, J. R. R., *M.C.* .. .. .. .. .. .. | 120 Henleaze Road, Henleaze. |
| *TRYON, VICTORIA S., M.B., Ch.B. Brist. .. | 3 Harley Place, Clifton, Bristol 8. |
| *WALKER, CYRIL H., B.A., M.B. Cantab. .. | Harewood, Clapton-in-Gordano, Som. |
| *WALTERS, C. F. .. .. .. .. .. .. .. .. | Downover, Walton Down, near Clevedon. |
| *WANSBROUGH, T. B., M.B., Ch.B. Brist. .. | 8 Mortimer Road, Clifton, Bristol 8. |
| WAKE, S. C., M.B., Ch.B. Brist. .. .. .. | Brantwood, Bideford, N. Devon. |
| *WARD, H. W. .. .. .. .. .. .. .. .. .. | Chipping Manor, Wotton-under-Edge. |
| *WARREN, R., M.A., M.D. Oxon. .. .. .. | Heathgates, Weston-super-Mare. |
| *WATERHOUSE, R,, M.D. Lond... .. .. .. | 3 The Circus, Bath. |
| *WATSON-WILLIAMS, E., *M.C.*, B.A., M.B., Ch.M. Cantab. | 65 Pembroke Road, Clifton, Bristol 8. |
| WATTS, J. B. V. .. .. .. .. .. .. .. .. | 6 Queen Street, Cambridge, C.P., South Africa. |
| WEDDELL, C. W. .. .. .. .. .. .. .. .. | Boydville, 46 Miller Street, West Melbourne, Australia. |
| WHITEHOUSE, Sir H. B., M.S. Lond. .. .. | 62 Hagley Road, Edgbaston, Birmingham. |
| *WIGODER, SYLVIA BEATRICE, M.A., M.D., Ph.D. | General Hospital, Bristol 1. |
| *WILBOND, R. G. .. .. .. .. .. .. .. .. | 92 Chesterfield Road, St. Andrew's, Bristol. |
| *WILLIAMS, I., M.B., Ch.B. Brist. .. .. .. | "Ardrem," Hill View, Henleaze, Bristol. |
| WILLIAMS, S. R., M.B. Lond. .. .. .. .. | Buckfastleigh, Devon. |
| *WILLWAY, F. W., M.D., M.S. Lond. .. .. | Bristol Royal Infirmary. |
| *WOOD, D. .. .. .. .. .. .. .. .. .. .. | 105 Pembroke Road, Clifton, Bristol 8. |
| *WOOD, W. V., *M.C.*, B.A. Cantab. .. .. .. | Henley Lodge, Yatton, Som. |
| *WOODMAN, DOROTHY, M.D. Lond. .. .. .. | Kynance, Downs Cote Park, Westbury-on-Trym, Bristol. |
| *WOOLLEY, A. W., M.B., Ch.B. Brist. .. .. | Melbourne House, Wells, Som. |
| *WOOLLEY, W., M.B., Ch.B. Brist. .. .. .. | 239 Cranbrook Road, Redland. |
| *WORTHINGTON, Lieut.-Col. F., M.B., Ch.B. Cantab. | Greytrees, Briercliffe Road, Coombe Dingle, Bristol 9. |
| *WRIGHT, A. J., M.B., B.S. Lond. .. .. .. | 2 Clifton Park, Clifton, Bristol 8. |
| *WRIGHT, WINIFRED M., M.B., B.S. Lond. .. | Merrymead, Shepton Mallet, Som. |
| *ZEALAND, L., M.D. Man. .. .. .. .. .. .. | Brecon Lodge, Westbury-on-Trym, Bristol. |

